# Changes in cortical brain activity after active break in preschoolers: MOVI-HIIT study

**DOI:** 10.3389/fspor.2025.1529288

**Published:** 2025-09-10

**Authors:** María Valdivieso-González, Francisco Javier Sancho-Bielsa, Beatriz García-Martínez, Arturo Martinez-Rodrigo, Oscar Navarro-Martínez, Andrés Redondo-Tébar, Mairena Sánchez-López

**Affiliations:** ^1^ Faculty of Education, University of Castilla-La Mancha, Ciudad Real, Spain; ^2^ Centro de Estudios Sociosanitarios (CESS), University of Castilla-La Mancha, Cuenca, Spain; ^3^ Faculty of Medicine, University of Castilla-La Mancha, Ciudad Real, Spain; ^4^ Polytechnic School of Cuenca, University of Castilla-La Mancha, Cuenca, Spain; ^5^ Research Group in Medical Computing, E-health and Advanced Technologies (COMETA Research Group), Universidad de Castilla La Mancha (UCLM), Cuenca, Spain; ^6^ Faculty of Nursing, University of Castilla-La Mancha, Ciudad Real, Spain

**Keywords:** electroencephalography, physical activity, children, acute effect, brain waves

## Abstract

**Introduction:**

This study investigates the acute effects of high-intensity interval exercise on brain activity in preschool children, focusing on changes in electroencephalogram (EEG) bands before, immediately after, and 20 min post-exercise.

**Methods:**

Eleven children (5–6 years) participated, but EEG recordings were included only if all channels maintained a quality score >3 (0–4 scale updated every 0.5 s), resulting in the exclusion of two participants due to insufficient high-quality data.

**Results:**

EEG data revealing significant increases in Theta, Alpha1, and Alpha2 bands, suggesting enhanced relaxation, attentiveness, and readiness to learn. Conversely, Beta and Gamma bands showed reductions, especially in frontal, temporal, and parietal regions, indicating decreased stress and mental effort.

**Discussion:**

These effects persisted for up to 20 min after exercise, suggesting that brief, high-intensity exercise induces EEG changes associated with states of attentional readiness and emotional regulation, which may support learning, pending further validation.

## 1 Introduction

According to the latest report of the World Health Organization, regular physical activity (PA) promotes and protects both physical and mental health in all population groups (1). Moreover, in children, several studies have shown that PA has a positive effect on cognitive and brain functioning (2–4). However, despite these benefits, the child population does not meet the minimum daily recommendations for healthy PA. Integrating PA into the classroom in the form of short periods of physical exercise (known as active breaks) not only offers students the opportunity to increase PA time but has also reported to be a simple and effective strategy for increasing attention in class (5–7).

Exercise triggers both acute (after a single bout of physical exercise, typically 5–40 min) and chronic (after several bouts of physical exercise per week over a prolonged period, typically more than six weeks) adaptations in the body. Short bouts of high-intensity PA led to immediate increases in the level of catecholamines, vasopressin and beta-endorphins in the peripheral circulation, which are thought to reflect increased neurotransmitters secretion in the central nervous system (CNS), leading to increased arousal and consequently improved cognitive performance (8). On the other side, the effects of chronic exercise occur through enhanced neurogenesis, neuronal connections (synaptogenesis), vascular development (angiogenesis) and metabolic factors related to brain function (neurotrophins) (9).

The effects of exercise on the brain can be analysed using an electroencephalogram (EEG), a procedure that measures the electrical activity of neurons in the cerebral cortex. This neurophysiological procedure evaluates neuronal function from an electrical perspective, using electrodes placed on the scalp. The procedure measures the electrical activity of neurons in the two hemispheres of the brain and their four lobes (frontal, temporal, parietal and occipital). Electrical activity is classified into different waves according to their frequency (Theta: 3.5–7.5 Hz; Alpha: 8–12 Hz; Beta: 13–30 Hz; Gamma: ≥30 Hz;) and these waves are associated with different situations in the individual (10).

Previous studies focusing on children older than 6 years, adolescents and adults have indicated that acute exercise has beneficial effects on cognitive function (11). Thus, a single session of acute exercise, at an intensity of 60% of maximum heart rate (HR) and lasting 20 min, has shown to improve the accuracy of responses on tests of cognitive control in children aged 9–10 years (12). Similarly, 20 min of moderate-intensity acute exercise has reported an increase of cortical activation in children of the same age (13, 14). These positive effects have also been observed in young adults, even 48 min after submaximal exercise at 83.5% of their maximal HR (14) and in children, an increase in Alpha activity was recorded 20 min after exercising at 65% of their maximum HR for 30 min on a cycle ergometer (15). However, neither the duration and intensity of exercise bouts, nor the duration of post-exercise electrical activity has been sufficiently elucidated in young children.

In a systematic review that included 11 studies of the acute effects of exercise on brain structure and neurophysiological function using EEG in children (16), only five found improvements in neuropsychological functioning (17–21), and only one of these was conducted in young children aged 5 years (18). However, these results contrast with the findings of a recent meta-analysis where no effect of acute PA interventions on different domains of cognitive function in preschool children (2–6 years) was found (22). Although it should be noted that in this meta-analysis the outcomes were general cognitive performance and not electrical activity as in the systematic review by Meijer et al. (16).

Given the paucity of studies on the acute effects of exercise on brain electrical activity in children, and given that the evidence on this subject is unclear, the present study examined changes in cortical electrical brain activity, by frequency band and region, using EEG between the rest state and immediately and 20 min after a 4-minute active break of moderate- to high-intensity HIIT-type interval exercise in 5- and 6-year-old schoolchildren.

## 2 Material and methods

### 2.1 Participants

Eleven preschoolers (6 boys and 5 girls) who were taking part in a MOVI-HIIT pilot trial (ClinicalTrials.gov NCT05243784) to test the effect of an active break's web platform (http://www.movihiit.es) in improving fitness, adiposity and executive function were selected. The students belonged to the third year of kindergarten in a public school located in the town of Ciudad Real province, Spain. They had a mean average age of 60,91 months (SD ± 6.95), a height of 110.24 cm (SD ± 5,93 cm), a weight of 19.16 kg (SD ± 3.52 kg), and a body mass index of 15.66 kg/m^2^ (SD ± 1.73 kg/m^2^). All participants had normotypical development.

### 2.2 Material, procedure and exercise protocol

The Emotiv EPOC headset and its software development kit for research mainly include 14 available channels: AF3, F7, F3, FC5, T7, P7, O1, O2, P8, T8, FC6, F4, F8, AF4 (also based on international locations 10–20) validated in young children (23). The impedance of the electrodes was reduced using saline liquid to the level required by the software. The Emotiv EPOC headset is a portable EEG device. Its lightweight design allows for use in various environments, making it especially useful in non-laboratory settings.

The parents of each participant read and signed an informed consent form, and each child gave verbal consent. The study protocol was approved by the Clinical Research Ethics Committee of the General University Hospital of Ciudad Real, Spain (reference number: REG: C-254).

The experimental procedure was applied during eight weeks, from March to April. EEG assessments were conducted during weeks 4 and 5 of the pilot study. Every day of the week, using the virtual platform MOVI-HIIT (http://www.movihiit.es), the children took two active breaks in the classroom during school hours. The active break was based on the high-interval training method and consisted of two blocks of four functional training exercises, such as “mountain climbers”, “jumping jacks”, “squats”, “skipping”, etc. Each exercise had a duration of 20 s followed by a rest of 10 s, for a total of 4 min of moderate to high intensity exercise. During the active break, their HR was recorded using photoplethysmography devices (Polar A300, polar H7 Hear Rate Sensor), with heart rate measured on the chest (average HR was 130 bpm).

**Figure 1:**
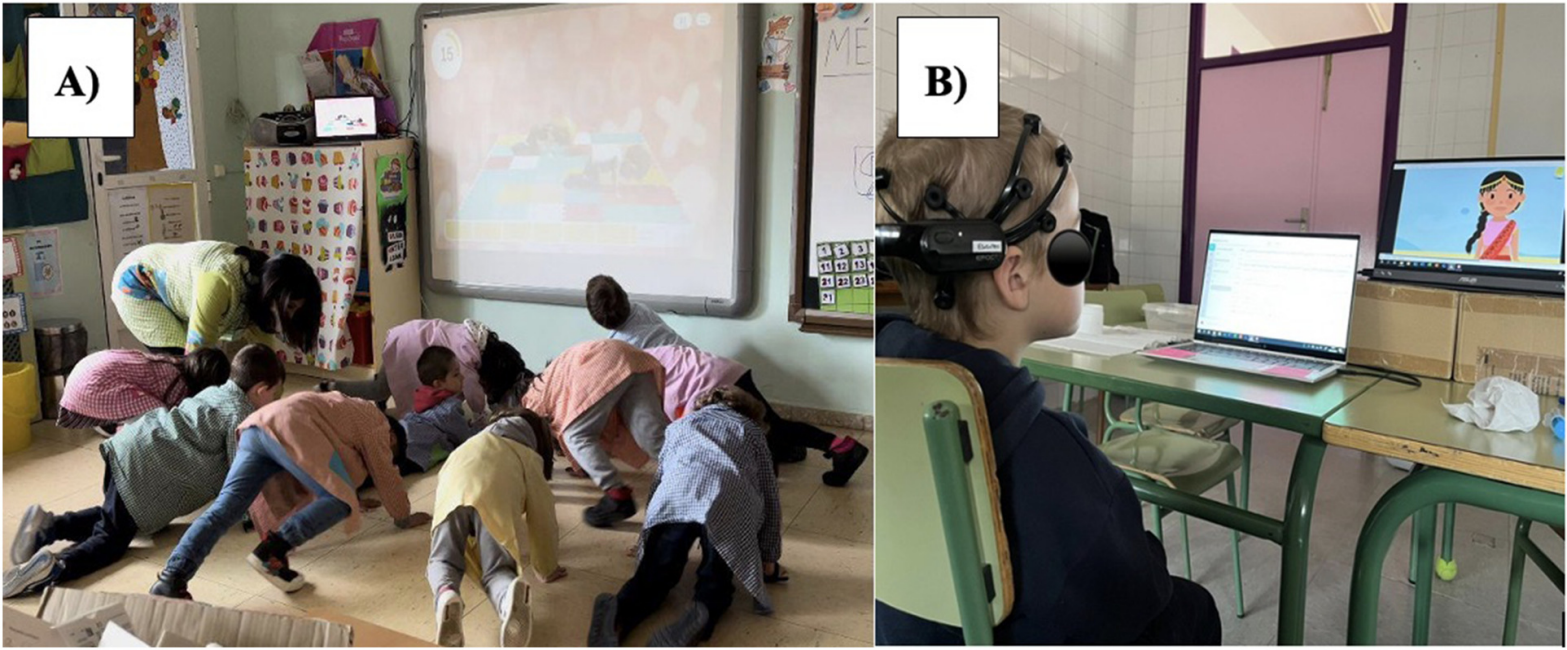
Active break and EEG recording. Active break and EEG recording. **(A)** Shows the active break performed in class through the MOVI- HIIT platform. **(B)** Shows the child with the Emotive EPOC headset visualizing the children's story.

**Figure 2:**
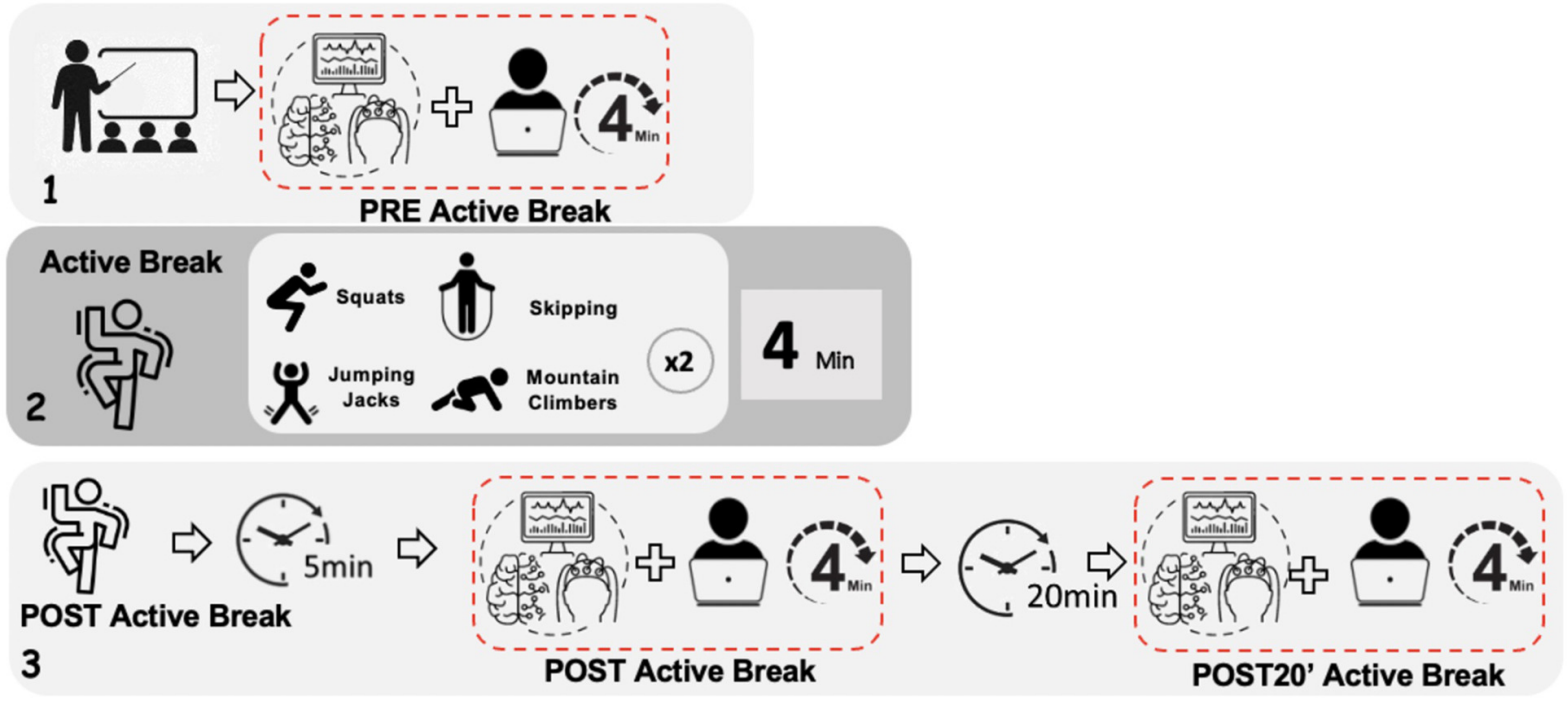
Protocol of the measurement. Box 1 corresponds to measurement 1. Box 2 corresponds to the active beak developing the exercises that were carried out. Box 3 corresponds to measurements 2 and 3.

The days of data collection, participants were seated in front of a computer in a room isolated from noise and connectivity interference; all mobile phones were set to aeroplane mode and the only network interference was from the two computers and the tablet that were used for EEG recording. Participants wore with the EMOTIV EPOC headset, and each computer recorded one subject, we took data from two subjects per day. Participants were asked to remain as relaxed as possible while looking at the computer screen. The centre of the PC screen was located at eye level, about 60 cm from the participants' heads. Subjects remained with their eyes open while watching a four-minute children's story (Figure 1). EEG recording with the headset was collected at three points in time: (1) before active break, (2) immediately after active break (recording started 5 min after the exercise) and (3) 20 min after active break. After the active break, which took place in the classroom, the subject was accompanied to the measurement room to continue with the second and third EEG measurement. All recordings followed the same protocol. Different video stimuli were used in POST and POST-20' assessments; however, both videos were matched for content, visual complexity, color scheme, and narrative structure to minimize stimulus-related variability. All three EEG recordings lasted the same length of time and followed the same conditions. The entire experimental session lasted approximately 2 h (Figure 2).

### 2.3 Selection and preprocessing of EEG signals

Emotiv EPOC+ provides information about the quality of the EEG signals registered during recording sessions, using a scale from 0 (minimum quality) to 4 (maximum quality) for each channel. This quality value is updated every 0.5 s. To ensure the quality of the EEG recordings analysed, only those segments from the original signals with all EEG channels having a quality value over 3 were finally selected, discarding those with poorer quality ratings. After this procedure, two participants were excluded for lacking enough high-quality data, resulting in nine participants included in the analysis. A summary table of signal quality criteria has been provided in Supplementary Material S1. They all presented good quality recordings in the three measurements, with durations between 5 and 300 s (average 185 ± 67 s). The remaining nine participants did not differ demographically from those who were excluded (*p* > 0.05).

Raw EEG signals selected were then preprocessed before the application of any kind of analysis method, thus noise and interferences were removed and only the information related to brain activity was maintained. This preprocessing step was carried out using EEGLAB, a Matlab toolbox specifically designed for the assessment and processing of EEG recordings (24). First, signals were re-referenced to the common average calculated as the subtraction of the mean potential of all channels from each single electrode (25). Then, two forward/backward high-pass and low-pass filtering approaches were applied at 4 and 45 Hz of cutoff frequency, respectively. These cutoff values allowed us to maintain the information of interest in emotional processes, included in Theta, Alpha, Beta and Gamma frequency bands (26). Simultaneously, these frequencies removed baseline and power line interferences from the EEG recordings. After that, some artifacts generated by either physical/physiological aspects (such as facial movements, eye blinks, or heart bumps) or technical causes (like electrode-pops or bad contacts of the electrodes over the scalp) remained in EEG signals. These remaining artifacts were eliminated using a blind source separation technique called independent component analysis (ICA). Briefly, it consisted of the decomposition of a signal into independent components, and those containing artifactual information were discarded, thus only maintaining the brain activity information.

### 2.4 Spectral feature extraction

The EEG signals from all participants in the three assessments were firstly divided into equally-sized segments of 5 s of length (i.e., 640 samples, as the sampling frequency of EMOTIV EPOC headset was 128 Hz). Hence, the total number of segments obtained for each assessment was N1 = 224, N2 = 260 and N3 = 212, respectively.

All the segments in the three assessments were analyzed from a spectral point of view by means of the computation of the power spectral density (PSD) for each EEG channel. It was calculated using a Welch's periodogram with a Hamming window of 2 s of length, 50% of overlapping and 256 points of resolution. Firstly, the spectral power of the whole band of interest (4–45 Hz) was computed for each EEG electrode as the area under the PSD curve between those frequencies:

$$SP_{\mathrm{all}}=\overset{45\;\mathrm{Hz}}{\underset{4\;\mathrm{Hz}}{\sum }}|\mathrm{PSD}(f)|$$

Then, the spectral power of each frequency band separately, i.e., SP_theta_, SP_alpha_, SP_beta_ and SP_gamma_, was computed similarly. However, the result for each band was normalized by SP_all_ in order to preserve the fluctuations among subjects:

$$SP_{\mathrm{band}}=\frac{1}{SP_{\mathrm{all}}}\overset{\;f_{2}}{\underset{\;f_{1}}{\sum }}|\mathrm{PSD}(f)|,$$

were f_1_ and f_2_ are the initial and final frequencies of the corresponding frequency band, i.e., 4–8 Hz for Theta, 8–10 Hz for Alpha1, 10–12 Hz for Alpha2, 12–30 Hz for Beta, and 30–45 Hz for Gamma bands.

### 2.5 Statistical analysis

With the purpose of verifying the effect of physical exercise in brain activity at short term, an inter-subject analysis of variance (ANOVA) was applied for the statistical comparison of the spectral activity of the brain before and immediately after the exercise (PRE vs. POST). A similar approach was developed to evaluate if the brain activity immediately after the exercise was maintained or changed after 20 min of exercise (POST vs. POST-20 min). For a further analysis of the effect of that resting period, a comparison between the brain activity before exercise and after 20 min of exercise (PRE vs. POST-20 min). These approaches were conducted for all EEG channels and all frequency bands, and only values of significance *ρ*<0.05 were considered as statistically significant. Given the number of statistical tests performed (14 channels × 5 frequency bands per contrast), a correction for multiple comparisons using the False Discovery Rate (FDR) method was applied in this updated analysis to reduce the risk of false positives. In addition, the results are also shown without the adjustment in Supplementary Material S2 and S3.

## 3 Results

**Figure 3:**
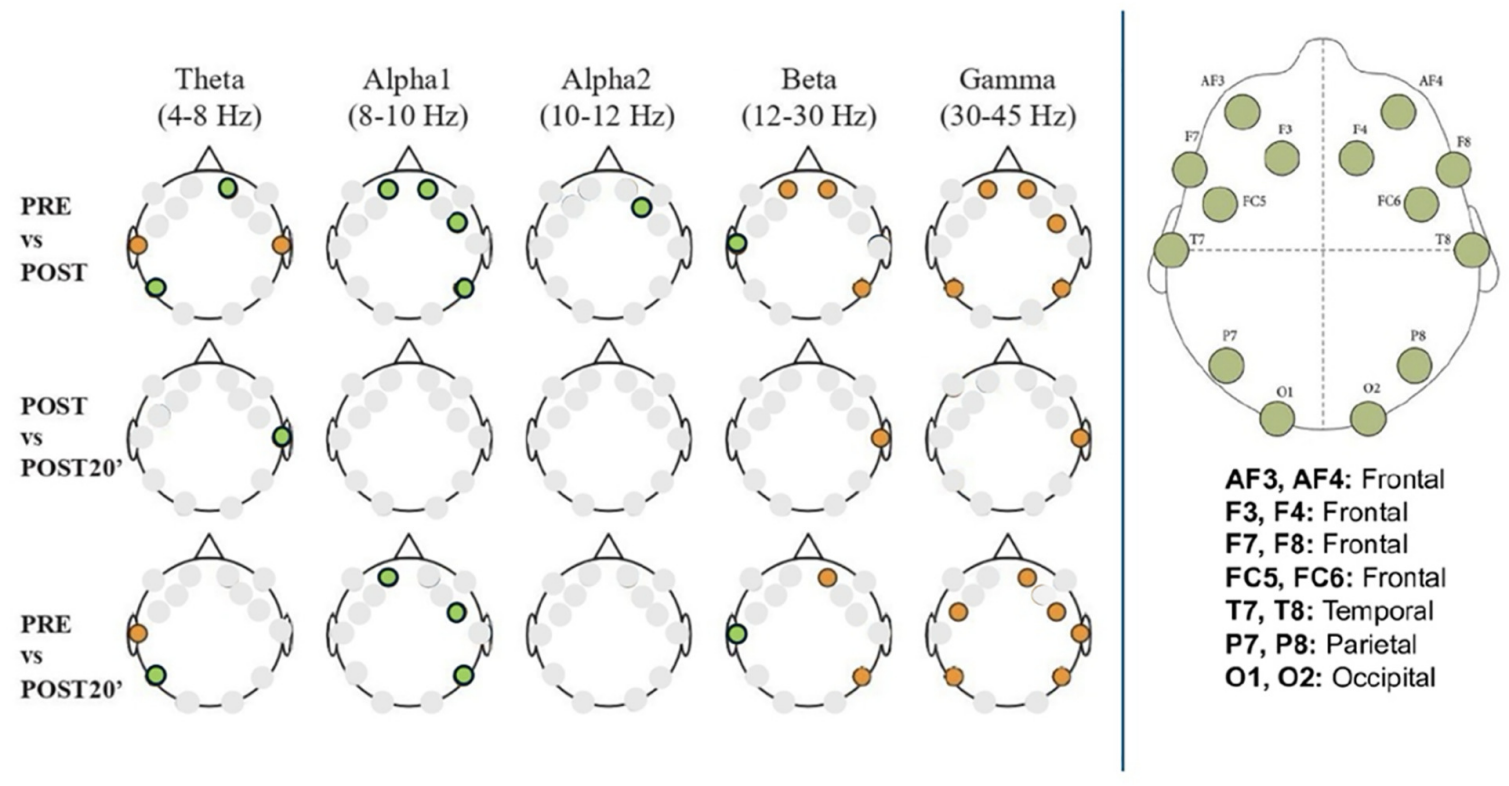
Cortical brain activity by frequency band across timepoints: PRE, POST, and POST-20 min. EEG spectral power is presented for each frequency band (Theta, Alpha1, Alpha2, Beta, Gamma) and compared across three timepoints: before the active break (PRE), immediately after (POST), and 20 min later (POST20). Comparisons include PRE vs. POST, POST vs. POST20, and PRE vs. POST20. Colored dots represent statistically significant differences (*p* < 0.05) at specific electrode sites, determined via inter-subject ANOVA models. Electrode positions correspond to the international 10–20 system. Orange dots indicate that power was significantly higher in the first condition of the comparison. Green dots indicate that power was significantly lower in the first condition of the comparison.

Cortical electrical activity is displayed in a heat map shown in the Supplementary Material S4. The Figure 3 shows a comparison of cortical brain activity before and immediately active break and 20 min later by frequency band and region using inter-subject ANOVA models. The results show that there were significant changes in brain electrical activation both from PRE to POST in Theta (AF4, T7, T8 and P7), in Alpha 1 (AF3, AF4, F4 and P8) in Alpha 2 (F4), in Beta (AF3, AF4, T7 and P8) and in Gamma (AF4, FC6 and P8), and from PRE to POST-20 min at in Theta (T7 and P7), in Alpha 1 (AF3, FC6 and P8), in Beta (AF4, T7 and P8) and in Gamma (AF4, FC5, FC6, T8, P7 and P8). The results also indicate that there were no relevant statistically significant differences between brain electrical activity immediately after exercise and 20 min after exercise, except in Theta (T8), in Beta (T8) and in Gamma (T8). Results without adjustment are shown in Supplementary Material S2.

**Figure 4:**
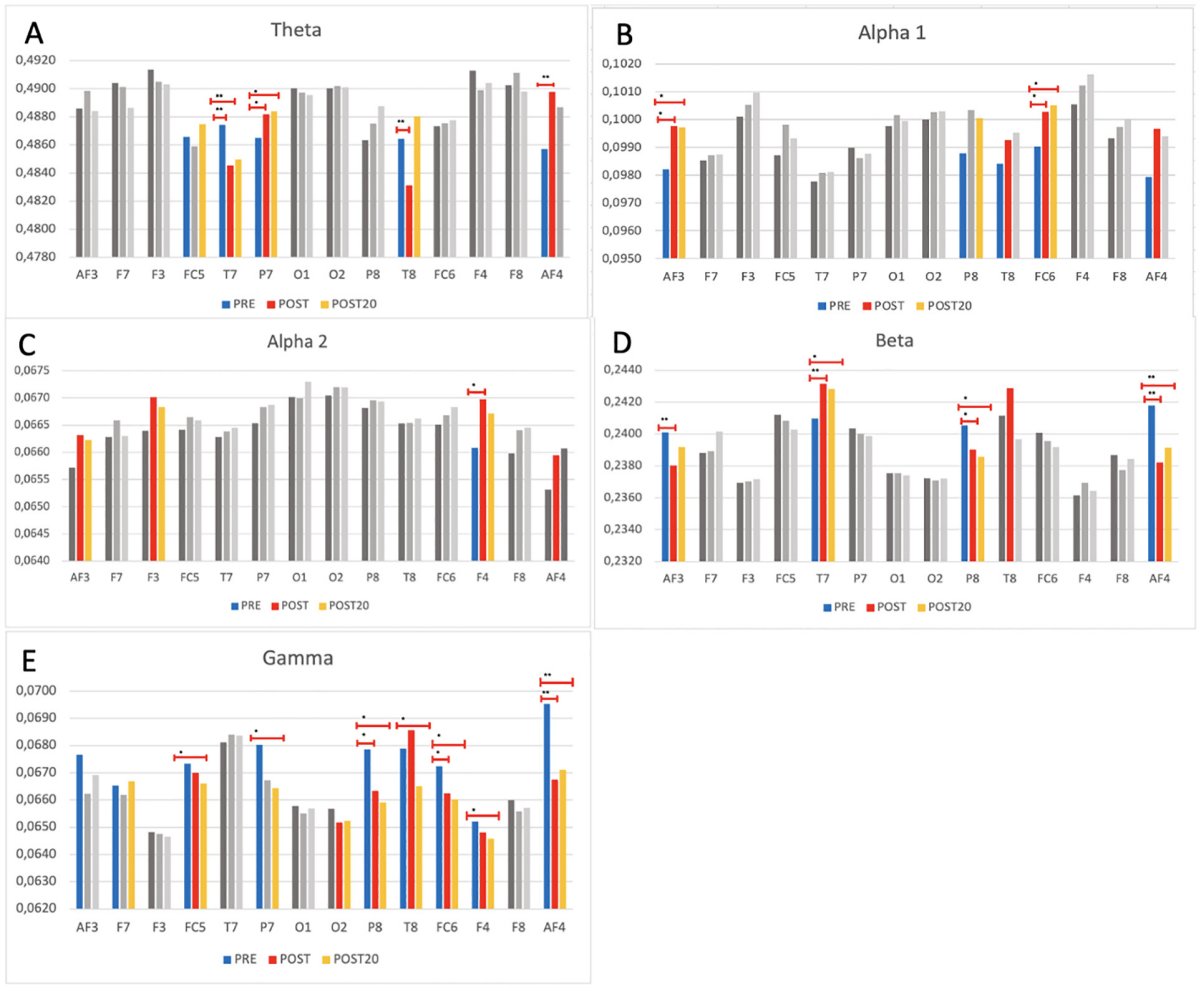
EEG spectral power across 14 electrode sites and five frequency bands before, immediately after, and 20 min after exercise. Bar plots represent mean EEG spectral power (± standard error) at each of the 14 electrode sites, across three timepoints: PRE-active break (blue), POST-active break (red), POST-20 min after break (orange). Data are presented separately for each frequency band (graph **A**: Theta, graph **B**: Alpha1, graph **C**: Alpha2, graph **D**: Beta, graph **E**: Gamma). Statistical analysis was conducted using inter-subject ANOVA with False Discovery Rate. Brackets indicate significant differences between timepoints (* denote *p* < 0.05 and ** donote *p* < 0.001).

Figure 4 shows the average power values in each frequency band, for each channel and for the three measurements: before and immediately after active break and 20 min later, using inter-subject ANOVA models. In brain activity Theta band, a decrease was observed in the central area (T7) after active break. As for brain activation after exercise, it was higher in the anterior and posterior channels (P7) and lower in the more central regions (T7 and T8). In terms of the effect of the passage of time post-exercise, the right central area showed the most activation, while the central and posterior regions (T7 and P7) were more activated after a period of 20 min post-active break (Figure 4A). Regarding the Alpha 1 band, brain activation was always lower before active break than after. As for the effect of the passage of time post-active break, the anterior channels (AF3 and AF4) were more active immediately after active break, while the central region (FC6) were more active after a period of 20 min post-active break (Figure 4B). In brain activity in the Alpha 2 band, brain activation before active break was always lower. Immediately after active break, an increase was observed in the anterior area (F4) (Figure 4C). As for the Beta band, decreases were observed between the pre-exercise measurement and the rest of the measurements in the anterior (AF4, AF3) and posterior (P8) regions. The central zone (T7) was more activated immediately after active break (Figure 4D). In brain activity in the Gamma band, all channels were shown to decrease from pre-active break to post-active break, except for the anterior zone at F7 where activation continued to increase after 20 min and the central zone (T8) where there was a large increase from pre-active break to post-active break, although this increase did not continue to rise after 20 min (Figure 4E). The supplementary material includes the results prior to adjustment (Supplementary Material S3).

## 4 Discussion

To the best of our knowledge, this is the first study to analyse cortical brain oscillations by frequency band and region after a bout of moderate to high intensity, short duration interval exercise in children aged 5–6 years. The results suggest an increase in the Theta, Alpha1, Alpha2 frequency bands and a decrease in the Beta and Gamma frequency bands immediately after the exercise and after 20 min compared to the resting state. These changes were mainly observed in the frontal, temporal and parietal areas, with a more pronounced tendency towards the right side of the head. In addition, our findings overall showed similar changes in brain oscillations immediately after exercise and after 20 min of exercise.

In general, our results indicate that after a 4-minute active break based on Moderate to Vigorous Physical Activity High Intensity Interval Training (HIIT) training, a significant increase in the oscillatory activity of the Theta, Alpha1 and Alpha2 bands is observed, accompanied by a decrease in the Beta and Gamma bands. These changes are consistent with EEG patterns previously linked to relaxed, alert states in which individuals remain awake and responsive, and which have also been associated in literature with reduced anxiety and stress. However, no direct behavioral or psychological measures were taken in this study, so this interpretation remains tentative. These findings are in line with previous studies in adults (27–30). In children over 8 of age, the review by Jimenez Vaquerizo (31) which analysed 23 studies on the effect of vigorous physical exercise on the P3 evoked potential and its wave amplitude using EEG, concludes that 4 of them reported an improvement in brain state, as evidenced by an increased electrical amplitude of the brain after exercise (32–35). However, it is not possible to directly compare the results of these studies with ours, as they did not analyse oscillations by frequency band, but focused on wave amplitude, so we should be cautious in their interpretation.

Our findings reveal increases in spectral powers in the Theta frequency band after exercise compared to the resting state in frontal (AF4) and parietal (P7) areas. According to Kochupillai (36) findings Theta waves (4–8 Hz) indicate that a person is experiencing a level of deep calm, similar to that experienced during sleep or meditation practice. These waves also serve as the basis for coordination between the senses and movement, as well as for the storage of spatially related information (37, 38). These results are consistent with those shown in the study by Crabbe & Dishman (39) in adults. This could indicate an improvement in attention and information processing capacity after exercise (40). Furthermore, the occurrence of oscillations in the theta band is associated with reward processing and active learning in early development (41).

Heightened frontal Theta oscillations recorded from frontal electrodes during cognitive control tasks (42, 43) and cognitive reappraisal, suggests the involvement of prefrontal structures in emotion regulation processes (44).

In contrast, our results show a decrease in this band in the temporal region (T7, T8), in line with the research by Fumoto et al. (45), which could be related to a shift in mental state towards greater alertness and activity oriented towards the external environment rather than internal reflection, which could affect attention and cognitive processing capacity after exercise (46, 47).

In line with our results concerning general cortical activation in the Alpha band (8–12 Hz), where activations are maintained after 20 min compared to PRE exercise, previous studies in children have found increases in Alpha power after acute exercise, using the EEG technique, which may reflect neural states consistent with reduced arousal (15, 18, 28). Additionally, Alpha brainwaves have been shown to be related to attentional demands, alertness, and expectancy (48). Also, in another study with badminton players, a sport that requires high concentration, attention and fast movements, an increase in Alpha waves was shown as an indicator of the level of attention of these athletes (49).

On the other hand, considering brain activity in Alpha according to brain region, increases were observed in the central lobe from PRE to POST and from PRE to POST- 20 min, suggesting that after exercise, children are likely to be in a state of attention, expectancy and vigilance, indicative of concentration processes (21). In the temporal lobe (T8) there are increases from PRE to POST and in the parietal lobe (P8) there are increases in both PRE to POST and PRE to POST- 20 min in Alpha1. In line with this, John & Schöllhorn (50) describe that in young adults, jumping on a skipping rope for just three minutes produces post-exercise increases in parietal Alpha waves, which are associated with favorable conditions for learning and cognitive performance.

While some authors such as Crabe & Dishman (39) found that exercise may increase Alpha activity, others reported minimal changes in frontal Alpha power following moderate exercise (51).

The Beta band (12–30 Hz) is present during normal waking consciousness, closely related to emotional, motor, perceptual and cognitive processes, reflecting complex mental and brain processes (52). Previous studies in adults have described that high-intensity exercise increases Beta frequency, associated with the release of catecholamines (53, 54). In contrast, and coinciding with our results, other studies have also found a decrease in Beta after physical exercise (28, 29). This decrease was evident in the frontal area (AF3, AF4) from PRE to POST and from PRE to POST- 20 min, in line with research by Brümmer et al. (29) in adults, which could indicate a state of relaxation or decreased mental effort after exercise.

In the temporal lobe, there are increases (T7) from PRE to POST active break and from POST to POST-20 min (T8). These results are in line with the research of Maureira Cid et al. (55) that after 30 min of intense physical exercise significant increases were seen in the Beta wave in the temporal region post exercise, translating into an improvement in working memory. In contrast, decreases were shown in T8 from PRE to POST-20 min, this decrease is related to the recognition of tones and content (visual content memory) fundamental in tests of sustained attention (56) as could be the visualization of the video in our study.

In the parietal lobe, decreases (P8) are observed in the measurement of PRE to POST and PRE to POST-20 min. This decrease in Beta could translate into a moment of calm, together with an improvement in concentration, reducing stress and anxiety after physical exercise.

In our results Gamma (30–100 Hz) shows decreases in all three measurements and in almost all brain areas. In line with research by Devilbiss et al. (51) where they described a reduction in Gamma activity in the left prefrontal cortex following acute intense physical exercise in adults, suggesting a cortical slowing, initiating a transition to a relaxed or calm state, and could ultimately be part of a process of functional reorganisation of the brain in response to exercise. In literature, Gamma waves are associated with higher-level cognitive processing, including the reception of novel information, senses, perceptions and states of happiness (57). In previous studies, Gamma wave activity has been found to be indicative of early information processing, selective attention, attentional direction, motivation and alertness (58). However, the paucity of studies analysing Gamma oscillations after exercise makes it difficult to compare our findings.

This post-exercise decrease suggests that the brain may be in a state of “recovery” or “relaxation”, where the need for high frequency processing decreases, allowing the brain to recover from fatigue. Again, due to the limited number of studies that have looked at Gamma activity, it is not possible to clarify why Gamma increases and decreases after exercise, which makes interpretation very difficult, especially in this age group (59).

### 4.1 Strengths and limitations

Although this study was conducted in a real environment in which schoolchildren are engaged in their academic activity (school), rather than in laboratory conditions, and in a population that has been poorly studied, which gives it added value, the results and their interpretation must be considered within their limitations. Firstly, the small sample size does not allow extrapolation of the results. Secondly, the measurement of brain oscillatory activity by EEG in young children presents a complexity that may make it difficult to obtain accurate and data. However, we believe that this bias was minimized by the standardization of the data collection process and rigorous data processing by experienced researchers. The selection of only high-quality excerpts from EEG signals diminished the length of the signals finally analyzed. However, only two participants were discarded, and the remaining subjects presented recordings with a proper length for its evaluation in the three measurements (PRE, POST and POST-20). Thirdly, although the 14-channel Emotiv EPOC EEG has been validated for use in young children and enables the measurement of cortical electrical brain activity (23), its spatial and spectral resolution is lower than that of clinical-grade EEG systems, which may limit the detection of subtle or regionally localized effects. While it is acknowledged that recording electrical brain signals in field studies is often impractical due to the specialized equipment and facilities required. The Emotiv EPOC headset offers a relatively low-cost, non-invasive, and portable approach for analyzing brain activity in real-world settings, such as school environments, albeit with reduced precision compared to more advanced EEG devices. Fourthly, examining oscillations after exercise without including a subsequent cognitive performance test means that no direct links to learning or emotional processes were measured or can be inferred. Therefore, interpretations regarding cognitive improvement or emotional changes remain speculative. Future research should address this limitation by incorporating direct measures of cognition and emotion into their experimental designs. Fifth, due to the small sample size and the limited number of segments per subject and condition, we could not account for between-subject dependencies in the statistical analyses. Consequently, segments were treated as independent observations, which may have inflated the degrees of freedom and increased the risk of type I errors. Sixthly, although the videos used in POST and POST-20' sessions were not identical, they were selected to be highly similar in terms of narrative, visual and auditory properties. Still, we acknowledge that the use of non-identical stimuli may introduce minor variability in EEG responses, and future studies should consider using fixed or counterbalanced stimuli to further control for this factor. Finally, it is important to consider the age of the participants, 5–6 years old, as the brain is not yet fully mature at this age, which could influence the results.

### 4.2 Conclusion

The results suggest that in children aged 5–6 years, immediately after and 20 min after a 4-minute active break of moderate to high intensity HIIT type interval exercise, oscillations in the Theta, Alpha1 and Alpha2 frequency bands increased, while those in the Beta and Gamma frequency bands decreased. These changes might be associated with states of relaxation in which participants remain awake and alert, as well as reduced levels of anxiety and stress. Future studies are needed to confirm these findings.

### 4.3 Practical implications

Based on the EEG changes observed, the following potential implications for classroom performance after active breaks are suggested: An increase in Theta waves has previously been associated with improved attention and information processing, potentially helping children remain focused during academic activities. Decreases in activity in the temporal region may reflect heightened alertness and external orientation, facilitating classroom engagement. Increased Alpha waves activity is consistent with a calm yet attentive mental state, which may support concentration and learning. Although these interpretations align with existing literature, they remain speculative in the absence of direct behavioral assessments in the current study. Conversely, a decrease in Beta, particularly in the frontal region, might reflect a reduction in mental effort and stress, potentially promoting a state of calm and improved concentration, which may support activities such as reading comprehension or problem solving. Moreover, a reduction in Gamma waves might be indicative of a shift toward relaxation and functional brain reorganization. This pattern could help children become calmer and less distracted, thereby potentially enhancing their ability to manage complex cognitive tasks.

## Data Availability

Data are available upon reasonable request from the corresponding author.
