# Supplementary material for: Changes in cortical brain activity after active break in preschoolers: MOVI-HIIT study
Source: Front Sports Act Living. 2025 Sep 10;7:1529288. doi: 10.3389/fspor.2025.1529288 (PMC12459121; doi:10.3389/fspor.2025.1529288)
Supplement: Supplementary file 1 [file Datasheet1.pdf]

Supplementary Material

Supplementary material 1

Table 1. Signal quality criteria

		s01	s02	s03	s04	s05	s06	s07	s08	s09	s10	s11
QUALITY 0-4	PRE	362,70	368,41	349,70	347,59	355,16	109,48	352,43	355,41	452,82	370,85	341,26
	POST	321,24	325,41	337,91	322,11	511,94	647,30	339,24	312,83	316,56	344,64	323,28
	POST 20'	305,58	306,20	305,53	338,96	306,12	206,00	304,55	294,37	297,05	348,20	308,17
QUALITY 3-4	PRE	299,00	3,00	189,50	151,00	6,00	5,50	263,50	163,50	333,00	67,00	177,00
	POST	243,39	0,00	185,50	163,50	3,00	178,00	247,00	206,50	214,00	154,00	172,00
	POST 20'	234,50	136,50	196,50	182,00	26,50	80,50	211,00	199,50	187,00	181,50	113,00

Note. *The following table presents the duration of the recordings for each participant and for each of the three measurements before and after the removal of the low-quality fragments, with FDR adjustment.*

Supplementary material 2

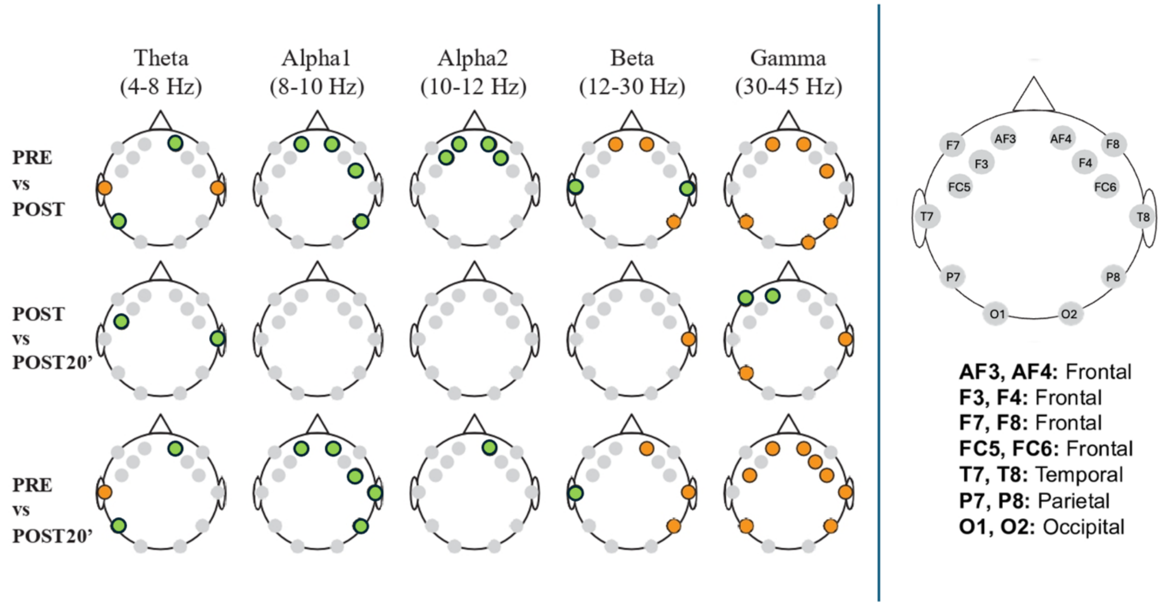

Figure 3. Cortical brain activity by frequency band across timepoints: PRE, POST, and POST-20 minutes. Without FDR adjustment.

Note. EEG spectral power is presented for each frequency band (Theta, Alpha1, Alpha2, Beta, Gamma) and compared across three timepoints: before the active break (PRE), immediately after (POST), and 20 minutes later (POST20). Comparisons include PRE vs POST, POST vs POST20, and PRE vs POST20.

Colored dots represent statistically significant differences (p < 0.05) at specific electrode sites, determined via inter-subject ANOVA models. Electrode positions correspond to the international 10–20 system.

- *Orange dots indicate that power was significantly* ***higher in the first condition*** *of the comparison.*
- *Green dots indicate that power was significantly* ***lower in the first condition*** *of the comparison.*

Supplementary material 3

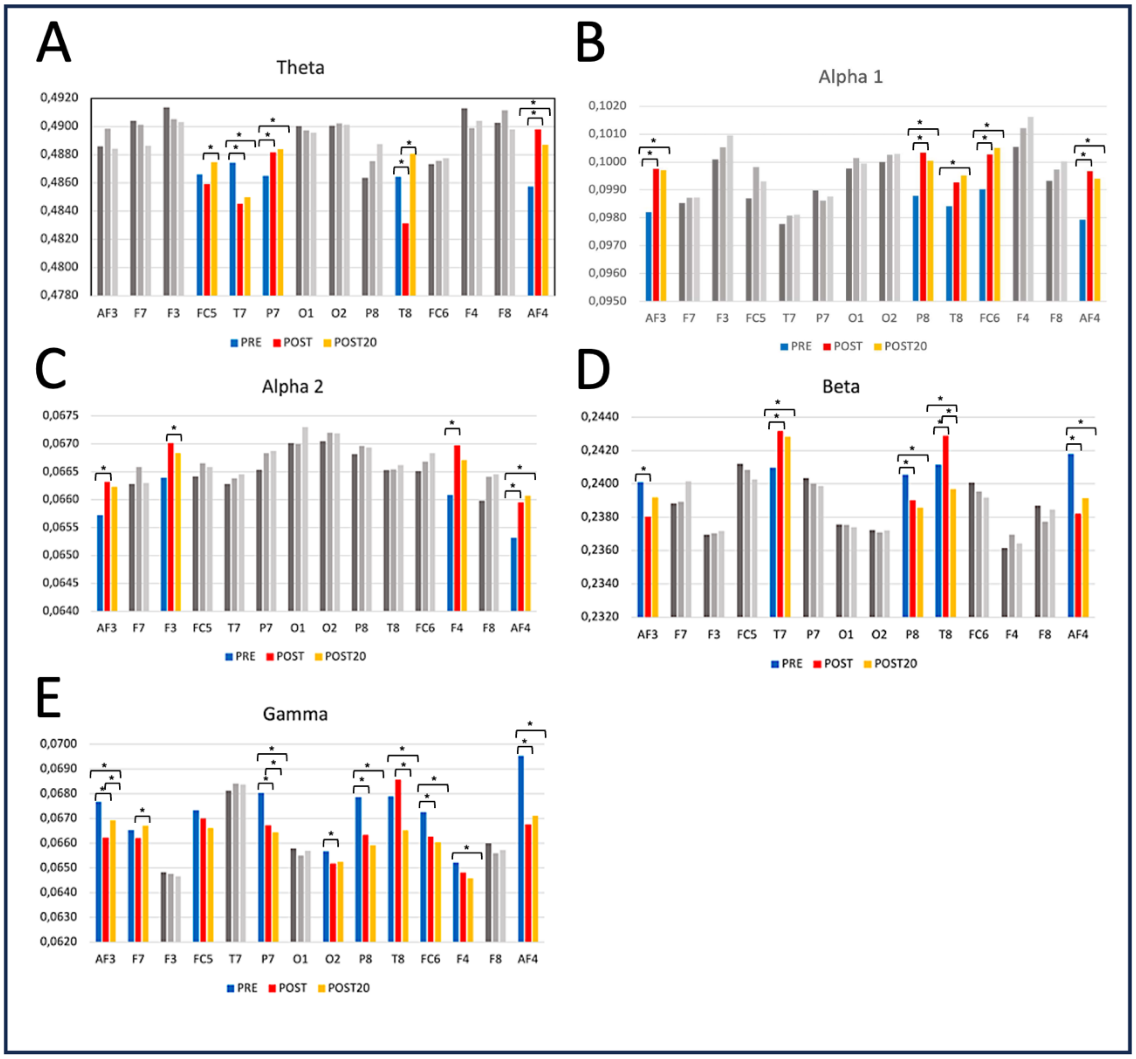

Figure 4. EEG spectral power across 14 electrode sites and five frequency bands before, immediately after, and 20 minutes after exercise. Without FDR adjustment.

Note. *Bar plots represent mean EEG spectral power (± standard error) at each of the 14 electrode sites, across three timepoints: PRE-active break (blue), POST-active break (red), POST-20 minutes after break (orange). Data are presented separately for each frequency band (Graph A: Theta, graph B: Alpha1,graph C: Alpha2, graph D: Beta, graph E Gamma). Brackets indicate significant differences between timepoints (* denote p < 0.05 and ** donote p < 0.001).*

Supplementary material 4

Figure 5. Cortical electrical activity is displayed in a heat map.

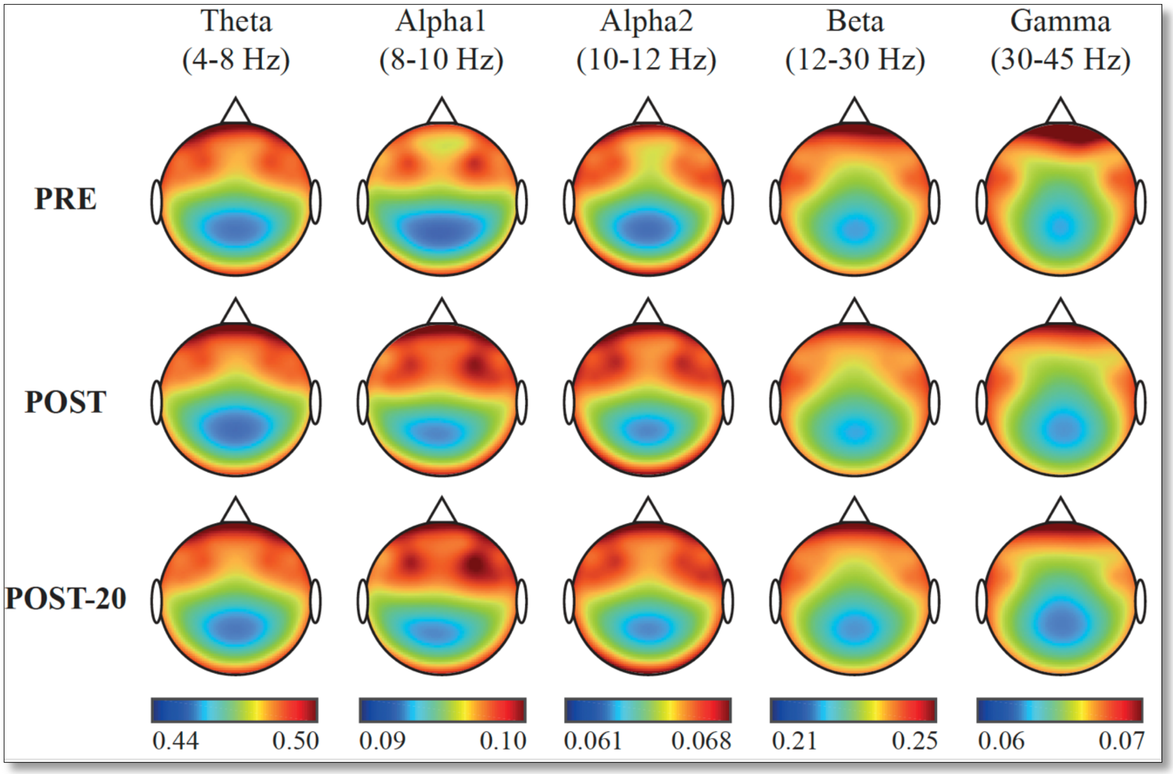

Note. *EEG spectral power is presented for each frequency band (Theta, Alpha1, Alpha2, Beta, Gamma) and compared across three timepoints: before the active break (PRE), immediately after (POST), and 20 minutes later (POST20). Comparisons include PRE vs POST, POST vs POST20, and PRE vs POST20 with FDR adjustment. Red and orange colors show the zones with higher activation and the green and blue colors show the zones with lower activation.*
